# Connecting Environmental Perception, Awe, Face Consciousness, and Environmentally Responsible Behaviors: A Mediated-Moderated Analysis

**DOI:** 10.3390/bs14070540

**Published:** 2024-06-27

**Authors:** Youxu Yan, Xueji Wang, Serene Tse, Lei Wang

**Affiliations:** Joint Institute of Ningbo University and University of Angers, Ningbo University, Ningbo 315211, China

**Keywords:** pro-environmental behavior, awe, tourist-environment perception, face, Pseudo Johnson–Neyman method

## Abstract

Tourists’ environmentally responsible behavior (TERB) is one of the key areas in tourism as it concerns tourism sustainability that further influences a destination’s competitiveness and image. This study sought to deepen the formation of TERB through tourists’ degree of environmental perception, mediated by their feeling of awe towards the environment, and moderated by their level of face consciousness. Data were collected from tourists in Tibet and the findings indicated that awe exerted partial mediation on the tourists’ perception-behavior relationship. Furthermore, face consciousness moderated the mediated relationship of humanistic environmental perception, awe, and TERB. This study adds value to existing tourism studies by revealing the influences of awe on constructing tourists’ attitude in sustainable tourism. From a practical perspective, the findings offer insights for destination management organizations (DMOs) in promoting tourists’ environmentally responsible behavior that fosters sustainable tourism development.

## 1. Introduction

Sustainable tourism has been a pressing and challenging topic among tourism academics as well as industry practitioners, and it depends on stakeholders’ attitudes which emphasizes how they perceive, feel, and act [1]. The increasing demand in tourism highlights tourists as one of the key stakeholders whereby their attitudes and behaviors could determine the success or even continuation of sustainable tourism within a destination. Among the various sustainable tourism challenges, environmental issues remain as one of the utmost priorities that are critical for tourism, ecology, as well as society [2,3]. The emphasis on the United Nation’s Sustainable Development Goals (SDGs) [4] fosters tourism to rethink and reshape tourists towards the environment which has been attracting the attention of tourism scholars. Tourists’ environmental responsible behavior (TERB) has become one of the important research topics.

The promotion of TERB calls on reducing harmful environmental impacts that were generated from tourists’ activities. More importantly, it focuses on tourist behavior to reduce negative environmental impact, contribute to environmental protection, and avoid disrupting the existing ecosystem or biosphere of the tourist destination [5]. TERB has been studied based on various theories and concepts such as the theory of reasoned action (TRA) [6], the theory of planned behavior (TPB) [7], value-belief-norm theory (VBN) [8,9], and the norm activation model (NAM; Pearce, Huang, Dowling, and Smith, 2022). These theories are effective tools for analyzing the process of the onset of human behavior and are empirically validated in various research areas.

The focus on TERB reflects the challenges of regulating tourist behavior in reality. Compared to everyday life scenarios, people’s behavior tends to be more indulgent in tourism settings [10]. Objectively speaking, there are fewer norms constraining individual behavior in tourism settings, as tourist destinations are always more tolerant of tourists due to the economic income they bring [11]. Subjectively speaking, individuals’ self-restraint also decreases. On one hand, this is because tourists in tourist destinations do not have long-term social relationships to maintain, which weakens their motivation to maintain a good self-image [12]. On the other hand, indulgence and pleasure are part of the motivation for many tourists to travel [13,14]. How to encourage tourists to overcome these influences and proactively practice environmental responsible behavior during their travels is one of the key issues faced by environmental protection in tourist destinations.

According to the stimuli-organism-response (S-O-R) model [15], an individual’s behavior is a response determined after processing external information in the brain. This inspires us to explore how to promote TERB, starting from tourists’ perception of the environment and the emotional changes triggered by it. Specifically, tourists’ behaviors are influenced by their perceptions and emotions whereby perception is generated from external stimuli while emotions are noted as the tourist’s internal states towards the external stimuli; eventually, tourist behavior is referred to as tourist’s response, forming the stimuli-organism-response (S-O-R) model. Moreover, He et al. pointed out that existing TERB studies lack of the emphasis on the psychological and emotional aspects of tourist behavior [16]. Examining TERB from the perspective of tourists’ perception and emotion will help to fill this research gap.

Furthermore, tourist’s behavior is constituted as a form of social behavior that could be influenced by the perception of others about them [17]. For instance, positive tourist behaviors are performed to establish a positive perception and image towards the tourists in the eyes of the others. Previous studies conceptualized such projection as face consciousness—a decency image with social responsibility [18]. While face consciousness is an important culture within the Chinese community, it should be noted that it is not limited to any single social group in guiding ethical judgement and moral practices. 

Overall, this research sought to contribute to the tourism literature in sustainable tourism and responsible tourists’ behaviors by examining the interrelationships between environmental perceptions, awe, TERB, and face consciousness. More importantly, it offers insights into how tourists’ perceived humanistic and natural environment may induce tourists’ degree of awe towards the environment which eventually affects their responsible behaviors. The findings of this research may offer insights for DMOs on promoting and managing pro-environmental tourist behavior through tourists’ emotional connections and their identity towards the environment.

## 2. Literature Review

### 2.1. Tourism in Tibet

Tibet has significant uniqueness in terms of natural environment, cultural customs, and social politics, which constitutes our reason for choosing Tibet as the case study. Firstly, in terms of the natural environment, Tibet is known as ‘the Third Pole of Earth’. It is located on the Qinghai-Tibet Plateau in southwest China, with an average altitude of over 4000 m. The unique meteorological and majestic geological landscapes such as snow-capped mountains and canyons in high-altitude areas easily inspire awe in tourists. Secondly, in terms of cultural customs, Tibet’s unique ethnic customs and the mysterious yet exotic Tibetan Buddhism also easily inspire awe in tourists. Lastly, in terms of social and political aspects, although Tibet being an inseparable part of China has become a consensus in the international community, the issue of Tibet’s legal status has once again become a fuse for ethnic conflicts under the influence of major power politics. On 14 March 2008, a violent incident against the Han ethnic group occurred in Lhasa [19], the capital of Tibet, which had far-reaching effects. Even today, many Han tourists (who are the main source of tourists in Tibet) still harbor fear about this, which undoubtedly adds a layer of mystery to Tibet. In addition, as tourism gradually becomes a pillar industry in Tibet, the impact of tourism activities on its fragile ecological environment has become a critical issue for all stakeholders [20]. Therefore, choosing Tibet as a case study also has significant practical significance.

### 2.2. Stimulus-Organism-Response (S-O-R) Model

The stimulus-organism-response (S-O-R) model was proposed based on the classic stimulus-response theory which further explains the relationship between extrinsic stimuli and individual behavior [15]. Stimuli trigger an individual’s state of cognition and affection, which then further exert an influence on the act of approach or avoidance [21]. Based on the S-O-R model, an individual’s action can be induced through two ways: (1) direct influence from cognition, forming the cognition-behavior relationships or (2) mediation through the presence of affection, forming the cognition-affection-behavior relationships. Stimuli can include both physical and psychosocial stimuli [22]. For instance, there are physical stimuli such as advertisement, delay [23], and psychosocial stimuli such as perceived severity, benefits, and barriers [24].

The S-O-R model has been applied and validated in various studies in a wide range of disciplines and contexts, including tourism [25,26]. Furthermore, Su and Swanson employed the S-O-R model to examine the interrelationship between perceived destination social responsibilities, consumption emotions, and environmentally responsible behavior [27]. Their findings revealed that stimuli post direct influence onto the response while consumption emotion severing as the organism mediated the direct effect of S-R. Base on the above, this study employed tourists’ environmental perception as the stimuli (S), the feeling of awe as the organism (O), and TERB as response (R), forming the environmental perception-awe-TERB model. 

### 2.3. Environmental Perception

Environmental perception refers to the process in which sensory organs collect information from the physical environment, which is then processed by the brain to guide the subject’s behavior; it can affect an individual’s cognition, emotions, and behavior [28]. The impact of environmental perception on individual behavior is a key focus of research. In the field of architecture, scholars have studied the impact of individual environmental perception in specific spaces on their behavior [29,30]. In environmental protection and ecology research, scholars have focused on the impact of environmental perception on ERB [31,32]. These studies have found that environmental perception has a significant impact on individual behavior.

Environmental perception of tourist destinations is the foundation and an important component of tourism activities, and it is also an important way for tourists to achieve tourism experiences. Its impact on tourist behavior has attracted the attention of scholars. Ryu and Jang conducted relevant research, measuring customers’ perceptions of dining environments in upscale restaurant settings from six aspects: facility aesthetics, lighting, ambience, layout, dining equipment, and employees [33]. They found that environmental perceptions impacted behavioral intentions through emotions (pleasure and arousal). The latest research has also concluded that environmental perception can significantly impact tourist behavior, especially TERB [34,35].

### 2.4. Awe

The concept of awe has been extensively studied in the fields of philosophy, sociology, and religion, with a focus on its connection to aesthetics, political change, and religion [36]. Keltner and Haidt’s initial research on awe pinpointed two primary cognitive appraisals: the perception of vastness and the mental necessity to integrate such vastness into existing mental schemas [37]. Noticing the scarcity of research on awe in tourism, Coghlan, Buckley, and Weaver created a framework for examining awe in tourism through a qualitative study [38]. This framework examined tourists’ awe experiences from a temporal perspective, encompassing an immediate physiological response, comparisons with previous experiences, and a forward-looking, schema-altering component. Powell, Brownlee, Kellert, and Ham outlined five subdimensions of awe in Antarctic tourism: the nature-human relationship, spiritual connection, transformative experience, goal clarification, and humility [39]. 

While the earlier tourism-related research tended to focus on describing awe-inspiring experiences using qualitative methods, later research aimed to explore antecedents and outcomes of awe using qualitative methods. Chen, Bai, and Luo found that awe impacts tourists’ helping behavior through time perception and the small self [40]. Yan, Shen, Ye, and Zhou established that the awe experience fully mediates the relationship between authenticity experience and experience quality [41]. Recent studies have found that awe, as a positive emotion, can significantly influence tourists’ behavior, including pro-environmental behavior [42].

### 2.5. Tourists’ Environmentally Responsible Behavior (TERB)

Against the backdrop of emphasizing sustainable development, there has been a growing interest in studying environmentally responsible behavior (ERB) as a crucial area of research, and tourists’ environmentally responsible behavior (TERB) is one of the important research directions [43,44]. ERB encompasses actions taken by individuals or groups to reduce negative environmental impacts, address environmental challenges, and/or find solutions to environmental issues [45,46]. Previous research on ERB has mainly focused on its antecedents. Based on the meta-analysis of the antecedents of pro-environmental behavior intention, Lin et al. categorized the main antecedents into five groups: ‘self-efficacy’, ‘affect’, ‘norms and values’, ‘environmental mindset’, and ‘place perceptions and evaluations’ [47]. 

Tourism activities rely on a good ecological environment in tourist destinations. Therefore, it is important to promote TERB in order to maintaining destination ecosystems. In the field of tourism, scholars have also conducted a few studies on the antecedents of TERB. Such as tourist environmental education [48], destination social responsibility [27], and hedonic and utilitarian values [49]. He et al. in their literature review on TERB proposed that future research on the onset of TERB could pay more attention to the role of psychological and emotional factors of tourists [16].

### 2.6. Face Consciousness

The notion of face, rooted in Chinese Confucian culture, serves as a subtle behavioral guide for Chinese individuals during social interactions [50]. The face represents a person’s self-image, which they can obtain from others within their social circle [51,52]. Qi characterizes the face as “the social anchoring of self in the gaze of others” [53]. Scholars from both China and the West have examined this concept, identifying two distinct aspects: *lian* and *mianzi* [53,54]. *Lian* pertains to one’s moral traits, representing the social esteem earned for moral uprightness and integrity. Losing *lian* signifies a total rejection of one’s moral character, it is unacceptable and often leads to shame and self-reproach [51]. *Mianzi* is associated with accomplishments, prestige, and social standing [55]. Losing *mianzi* highlights the damage to one’s positive social reputation in the eyes of others, causing embarrassment [56,57]. However, losing *mianzi* is not as intolerable as losing *lian*.

In China, tourists not only fear losing *lian*, which symbolizes their moral level, but also fear losing *mianzi*. This is because although tourism has become more popular in China with the improvement of the economic level, tourism is still a privilege of higher income groups due to the uneven economic development levels between regions and between urban and rural areas [58]. Therefore, tourists represent a higher social status, especially for those tourists who travel from developed to less developed areas. They often believe that they are “more civilized” than the locals and do not want their civilized image to be destroyed by their uncivilized behavior. The impact of face consciousness on behavior is the focus of face research. In management, there is a large amount of research that empirically demonstrated the impact of face consciousness on individual behavior (especially consumer behavior), such as its impact on high-carbon consumption behavior [59], on energy-saving behavior [60], and on luxury consumption behavior [61], etc. Many of these studies have shown that face consciousness significantly affects individuals’ ERB. In the tourism context, there are also some studies that have verified that face consciousness can affect TERB [62,63].

## 3. Hypotheses Development

### 3.1. Environmental Perception and Awe

Shiota, Campos, and Keltner found that vast and spectacular scenes, such as mountains and highly sacred temples, are primarily likely to induce awe in tourists [64]. Previous research has found that tourists’ natural and religious environmental perception of mountain type tourism destinations induces tourists’ awe [65,66]. While in Tibet, besides Tibetan Buddhism (a religion popular in Tibet), its high-altitude geographical environment and nomadic lifestyle have created other unique and mysterious cultural landscapes in the eyes of tourists. Therefore, this paper assumed that it is tourists’ humanistic and natural environmental perception that stimulate their awe and proposed the following hypotheses:

**Hypothesis** **1a.** *Tourists’ humanistic environmental perception impacts the feeling of awe positively*.

**Hypothesis** **1b.** *Tourists’ natural environmental perception impacts the feeling of awe positively*.

### 3.2. Environmental Perception and Tourists’ Environmentally Responsible Behavior (TERB)

Regarding S-R theory, Watson posited that all human behavior can be interpreted as a response to a stimulus [67]. The tourist is stimulated by the destination environment, and through physiological and psychological reactions and feelings, tourists form perceptions of the environment and make corresponding behavioral responses. Qiu, Wang, Ren, Zhang, and Wang confirmed the implications of environmental perception on TERB through empirical research [68]. Therefore, we argued that environmental perception positively influences TERB. Thus, the following hypotheses were proposed:

**Hypothesis** **2a.** *Tourists’ humanistic environmental perception impacts TERB positively*.

**Hypothesis** **2b.** *Tourists’ natural environmental perception impacts TERB positively*.

### 3.3. Awe and Tourists’ Environmentally Responsible Behavior (TERB)

Qi et al. found that TERB is directly and indirectly (via place dependence) influenced by awe [66]. Wang and Lyu confirmed that by reducing the focus on the individual self, inducing awe through tourism experiences may promote ERB [42]. In addition, Yan and Jia confirmed that awe potentially influences TERB through mediators (i.e., new ecological paradigm, awareness of consequence, ascription of responsibility, and personal norm) [69]. Accordingly, the following hypothesis was developed:

**Hypothesis** **3.** *Tourists’ feeling of awe impacts TERB positively*.

### 3.4. Mediating Role of Awe

Previous empirical studies have argued that awe and anticipated self-conscious emotions can play a mediating role between embodied perceptions and tourists’ environmentally responsible behavior intentions [70]. In our model, environmental perception works as a stimulus by inspiring tourists’ awe, which in turn promotes TERB. Based on the stimulus-organism-response framework and previous empirical findings, we hypothesized the following:

**Hypothesis** **4a.** *Tourists’ feeling of awe mediates the direct effect of humanistic environmental perception onto TERB*.

**Hypothesis** **4b.** *Tourists’ feeling of awe mediates the direct effect of natural environmental perception onto TERB*.

### 3.5. Moderating Role of Face Consciousness

The moderating role of face consciousness on the relationship between behavior and its antecedents has been confirmed in previous studies. Wu, et al. determined that the fear of losing face moderates the effect of personal norms on ERB [18]. Zhao, Bai, Liu, and Wang revealed that *mianzi* moderates the effect of green self-identity on purchase intention [71]. In addition, Shan, et al. validated that the face moderates the effect of brand social power on intention to purchase [61]. Accordingly, the following hypotheses were developed:

**Hypothesis** **5a.** *Tourists’ level of face consciousness positively moderates the direct effect of humanistic environmental perception onto TERB*.

**Hypothesis** **5b.** *Tourists’ level of face consciousness positively moderates the direct effect of natural environmental perception onto TERB*.

**Hypothesis** **6a.** *Tourists’ level of face consciousness positively moderates the indirect effect of humanistic environmental perception onto TERB through the feeling of awe*.

**Hypothesis** **6b.** *Tourists’ level of face consciousness positively moderates the indirect effect of natural environmental perception onto TERB through the feeling of awe*.

**Figure 1:**
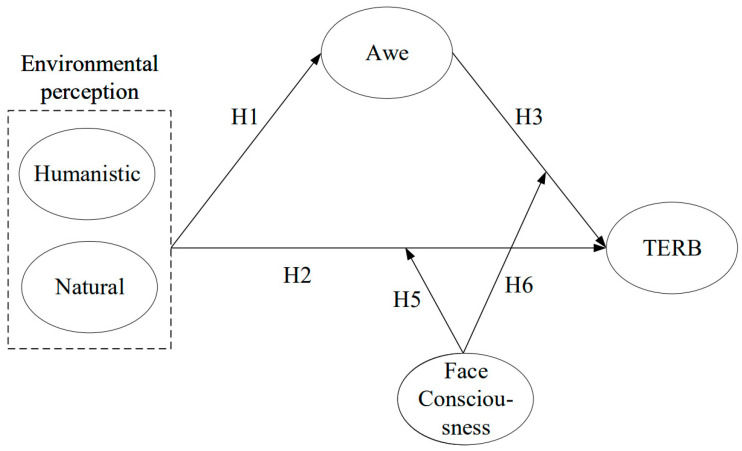
Proposed hypotheses and model.

Figure 1 shows the research model based on the above hypotheses.

## 4. Research Methodology

### 4.1. Questionnaire Design

Various scales were employed to assess the humanistic environment perception, natural environment perception, awe, face consciousness, TERB. More specifically, humanistic and natural environmental perceptions were examined using six and three questions respectively, adapting from Tian, et al.’s work [65]. Seven questions were used to examine the concept of awe proposed by Tian, et al. [65] and Coghlan, et al. [38] while seven questions from Zhang and Bai were adopted to measure face consciousness [72]. Finally, TERB was measured based on the four items of Cheng et al. [73]. All items, except for awe questions that were examined using a 5-pointed differential scale, were measured using a 5-point Likert scale ranging from 1 = strongly disagree to 5 = strongly agree. The items for each construct can be found in Appendix A.

All questionnaire items were presented in Chinese, and the only English scale, TERB, was translated into Chinese before synthesizing with the other measurement items. A back-to-back translation was performed on the four measurement items and were reviewed by the research team to ensure the meaning was retained. Additionally, experts were invited to review the questionnaire, provide suggestions and modification accordingly. The final questionnaire was administrated in Chinese. 

### 4.2. Data Collection and Sample

**Figure 2:**
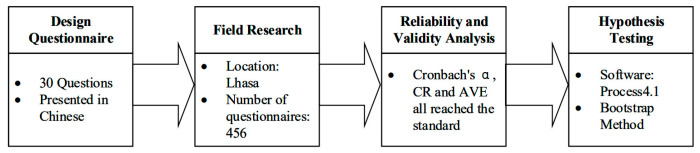
Process of data collection and analysis.

Data was collected offline and on-site to a targeted sample of 500 tourists who arrived at Lhasa, the most popular tourist attraction and the transportation hub of Tibet [74,75]. These tourists were all Chinese domestic tourists, and individual travelers accounted for 95%. Given the inability to obtain the gender ratio of tourists in Tibet from public data, this study adopted a 50:50 gender-quota sampling approach, referencing the results of random sampling surveys of Tibetan tourists in previous research [76], which showed little difference in the male to female ratio. The research team intercepted tourists, explained the research objectives and assured data confidentially as well as the right to terminate the study at any time. Tibetan handicrafts such as bracelets and prayer wheels were given as tokens of appreciation to the respondents. The process of data collection and analysis is shown in Figure 2.

Eventually, 456 completed surveys were collected, resulting in a response rate of 91.20%. Forty-four questionnaires were discarded due to incompleteness. A total of 62.90% of the respondents were aged between 20 to 39 years old, 78.70% had attained at least a university degree, and 40.80% earned less than RMB4000 per month.

To assess the risk of common method bias (CMB), Harman’s single-factor test [77] was performed. The results of an exploratory factor analysis with all the variables set at one unique factor showed that the largest (first) factor accounted for 31.879%, lower than 50% [78]. Thus, CMB was not a critical concern in this study.

## 5. Results

Confirmatory factor analysis (CFA) was conducted to assess the measurement model (Table 1). The measurement model fitted the data well (CMIN/DF = 3.242, SRMR = 0.0429, RMSEA = 0.070, GFI = 0.859, IFI = 0.911, TLI = 0.900, and CFI = 0.910). The composite reliability (CR) value for each dimension ranged from 0.759 to 0.950, exceeding the threshold value of 0.7, representing good internal consistencies. Convergent validity was achieved as all dimensions had an average variance extracted (AVE) above 0.50, ranging from 0.506 to 0.729. Furthermore, discriminant validity was achieved (Table 2) since all dimensions had an inter-dimension correlation coefficient lower than 0.85 and less than the square root of the AVE [79].

### 5.1. Mediation Model

**Figure 3:**
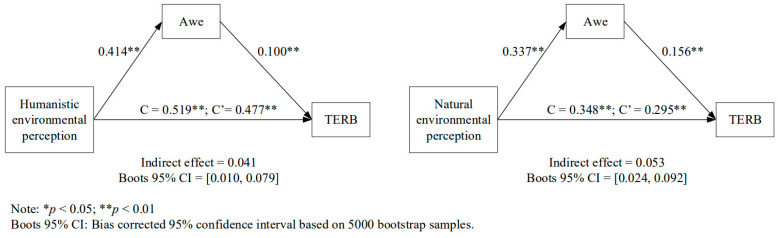
Results of mediation model.

Proposed mediation was performed on environmental perception (humanistic and natural), awe, and TERB using SPSS PROCESS v3.4 model number 4 with 5000 bootstrap sample at 95% confidence level [80]. The results provided empirical evidence for the interrelationships for the proposed models as environmental perceptions affected an individual’s awe and their TERB in the tourism setting. More importantly, the results revealed the mediation effect of awe on the direct effect of environmental perceptions and TERB (Figure 3). 

Specifically, positive direct effects were achieved among the independent variables and mediator and dependent variable. Interestingly, humanistic environmental perceptions, as compared to natural environmental perceptions, posted higher influences towards both awe and individual’s TERB. The results provided evidence in support of H1, H2, and H3. 

The results indicated awe mediated partially the direct effect between an individual’s humanistic environmental perception and TERB (indirect effect = 0.041; boots 95% CI = [0.010, 0.079]). Furthermore, awe also posted a partial mediation effect on the direct relationship between natural environmental perception and TERB (indirect effect = 0.053; boots 95% CI = [0.024, 0.092]). The findings supported H4a and H4b.

### 5.2. Moderated Mediation Model

In order to test the moderation effect of tourists’ face consciousness on paths c and ab, namely: (1) direct effect between humanistic/natural environmental perception towards TERB, and (2) indirect effect between humanistic/natural environmental perception towards TERB, of the hypothesized mediation model, PROCESS Model 15 with 5000 bootstrapping at 95% confidence level [80] was run to determine Hypotheses 5–6. The results are shown in Table 3.

The results revealed that no moderation effect was found between the direct effect of natural environmental perception and TERB; H5b was therefore not supported. However, significant and positive moderation was noted on the direct effect of humanistic environmental perception and TERB, thereby supporting H5a. 

Hypothesis 6 (a and b) stated the conditional indirect effect, via awe, of both humanistic environmental perceptions (H6a) and natural environmental perceptions (H6b) on TERB are positively moderated by an individual’s face consciousness. As shown in Table 4, the bootstrap confident level of the index of moderated mediation for hypothesis 6b but not for 6a crossed zero, meaning that the indirect effect of environmental perceptions (only humanistic but not natural) and TERB via awe varied according to the level of face consciousness of tourists. However, this variation was in the opposite direction, but the moderating effect was positive in Hypothesis 6a, hence rejecting Hypotheses 6a and b. 

**Figure 4:**
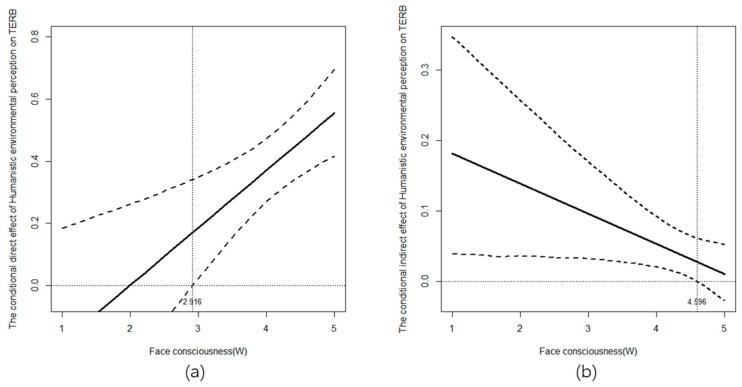
The conditional direct (**a**) and indirect (**b**) effect of humanistic environmental perception on TERB as a function of face consciousness. Note: The two dotted lines represent the upper and lower bounds of the confidence interval.

Furthermore, Johnson–Neyman and Pseudo Johnson–Neyman methods [80] were employed to detect the conditional direct and indirect effect of humanistic environment perception on TERB respectively (Figure 4). Specifically, when using the Pseudo Johnson–Neyman method, we took 10,000 values evenly from one to five (the value range of face consciousness) and visualized the coefficients and significances of the indirect effects at these values. As shown in Figure 4a, the direct effect of humanistic environment perception on TERB became positive and significant only when face consciousness was greater than 2.916. Among all respondents, the percentage of face consciousness value below 2.916 was only 3.290% while the percentage above 2.916 was 96.711%, demonstrating that in most cases, this direct effect was significant and positive. As shown in Figure 4b, only when the value of face consciousness was less than 4.596, the indirect effect of humanistic environment perception on TERB through awe emotion was positive and significant. Among all respondents, 57.456% had a face consciousness score of below 4.596, and 42.544% were higher than 4.596, demonstrating that only when the face consciousness was at a relatively medium-low level (still a high level in an absolute sense), the indirect effect was positive and significant, while it was insignificant when the face consciousness was at a relatively high level.

## 6. Discussion and Conclusions

### 6.1. Discussion

This research examined the influences of tourists’ degree of awe towards the environment as the mediator and face consciousness as the moderator on the relationship of tourist’s perception and TERB. Findings indicated environmental perception, both humanistic and natural, exerted positive impacts on tourist’s emotional of awe, corroborating existing studies that suggest awe is a positive emotion that is determined by an individual’s perception towards the destination’s humanistic and natural environment [65,66,70]. Furthermore, the findings echoed existing studies that provide empirical evidence in the establishment of a tripartite model of environmental perception, emotion of awe, and TERB, representing stimuli, organism, and response, respectively, and forming the S-O-R model of TERB [42,69,81]. Moreover, the findings also revealed the moderation effect of tourist’s level of face consciousness in the tripartite model.

However, the present study also found that face consciousness had no significant moderation effects when natural environmental perceptions were serving as the independent variable. One of the possible reasons could be because face consciousness is rooted in the interaction between individuals and focuses on the individual’s perception of self and others in the inter-group environment [55,62,82]. In other words, face consciousness plays a role in the context of social interaction that is situated in the context of humanistic environmental perception which is often socially oriented instead of in natural environmental perception that is nonsocial. Therefore, face consciousness fails to moderate the direct and indirect influence between natural environmental perception and TERB. Another possible reason could be that tourists were overwhelmed and immersed in the ‘awe-shock’ brought about by the majestic and magnificent natural landscapes of Tibet that neglected the attention on face consciousness. While in the case of humanistic environmental perception, social setting remained dominant and may have fostered tourists’ emphasis on their level of face consciousness.

Another interesting finding was face consciousness exerted a negative moderation effect on the indirect effect of the tripartite model significantly. A possible explanation could be the competing effect between awe and face consciousness in eliciting TERB from the humanistic environmental perception. For instance, in an individual with a stronger face consciousness, the psychological fear of losing face when engaging in environmentally unfriendly behavior will hinder them from committing such acts. Also, such a psychological fear overshadowed the emotion of awe, with awe’s role in behavior regulation masked by face consciousness.

Finally, as mentioned in the introduction, previous studies on the formation of TERB have overlooked the psychological and emotional aspects of tourist behavior, which were precisely the key factors that motivate tourists to actively practice environmentally responsible behavior. Taking this study as an example, if tourists form some emotional connection with the tourist destination through a series of tourism activities, such as feeling awe for the destination or some of its elements, then tourists will subjectively want to protect it and contribute to it, and naturally, they will not have behaviors that cause damage to it. Moreover, when tourists realize that they are under the scrutiny of others, if they perform uncivilized behaviors such as littering, they will lose their face, then even without the supervision of management personnel, tourists will actively regulate their behavior. Therefore, in future academic research and tourism destination management practices, the use of psychological and emotional factors to stimulate tourists’ active environmentally responsible behavior should receive more attention.

### 6.2. Conclusions

This study fills the gap in the existing literature on TERB that lacks attention to the psychological and emotional aspects of tourist behavior. Based on the S-O-R model, a triangular model including environmental perception, awe, and TERB was constructed, and the moderating effect of face consciousness was examined. Empirical research results showed that tourists’ environmental perception can directly and indirectly (through inducing awe emotion) influence their TERB, and this process was affected by the tourists’ own face consciousness. This study successfully validated the impact of two psychological and emotional variables, awe, and face consciousness on TERB, providing a theoretical basis for stimulating proactive TERB using psychological and emotional factors.

## 7. Implications

### 7.1. Theoretical Implications

This research offers several theoretical contributions. Firstly, it provides empirical evidence that emotion of awe serves a mediator in the direct relationship between environmental perception and TERB. Such findings compliment with the concept of the S-O-R model of tourists’ perception-behaviors relationships whereby the environmental perception of the tourist destination (S) induces awe in the tourist (O), which in turn promotes TERB (R). Specifically, this research integrated existing English and Chinese literature that focused on environmental perception, emotion of awe, responsible behaviors, and face consciousness in the tourism context [43,63,70]. In doing so, this research revealed the interrelationships amongst tourists’ humanistic and natural environmental perceptions, emotions of awe towards the destination’s environment, their TERB. Furthermore, the moderation effect of face consciousness onto the formation of TERB.

Next, this study also contributes by indicating tourists’ level of face consciousness as a moderator in the construction of TERB. In the context of this research, it required a specific preliminary condition for activating the moderating effect of face consciousness, for example, the tourist is placed in the context of social interaction. This revealed the conditional boundary of face consciousness towards the elicitation of TERB under different forms of environmental perceptions. This echoes the work of Li and Cui, which found that compared with the individual situation, face consciousness had a stronger impact on the purchase intention when the individual was in a group situation [83] and inspires us to consider the conditions under which face consciousness plays a role when we use it as an antecedent variable to explain behavior; the conditions are also worthy of further study.

Furthermore, the findings also highlighted the possible competition between emotion of awe and tourists’ face consciousness in the formation of TERB. Additionally, face consciousness has overshadowed awe in the process of producing effects on TERB. This inspires us to pay more attention to the interaction between antecedents when studying the formation of a certain behavior. Especially for TERB, a behavior that has received wide attention from scholars, we have identified many antecedents of TERB through previous studies. The next research may focus on the competitive or complementary relationship between variables to deepen the understanding of the formation of TERB.

### 7.2. Practical Contribution

This study also provided the following insights for destination management organizations (DMOs). Firstly, based on the mediating effect of the emotion of awe, it suggested that tourism managers and industry practitioners should focus on creating this emotion among the tourists when they are participating in tourism activities. These activities may include adventure activities such as bungee jumping, skydiving, or white-water rafting, wildlife encounters such as whale watching, or bird watching, and cultural experiences such as participating in traditional performances, workshops, or festivals that showcase the local heritage and customs. These activities can induce tourists’ respect and emotional attachment with the humanistic or natural environment, enhancing their TERB and reducing harmful impacts towards the environment.

Secondly, the finding of this study found that face consciousness moderated TERB formation. It suggested that tourism destination management could utilize face consciousness as a tool to regulate tourist behavior. Face consciousness plays a role in the context of social interaction. Therefore, it is essential to keep tourists aware that they are constantly being compared with other visitors, especially strangers, and are under their scrutiny. To achieve this goal, on one hand, interaction between strangers should be encouraged. People usually want to make a good first impression on others, so they pay more attention to regulating their behavior. On the other hand, gentle reminders such as public sign boards with notes of appreciation to tourists for not disturbing wildlife would guide tourists to compare their own behavior with the civilized behavior of previous tourists, so as to regulate their own behavior. Other reminders could also be about monitoring others’ ethical behavior, such as ‘Is the person next to you throwing garbage into the rubbish bin?’ These reminders could be posted at public resting places (e.g., on hiking trails) to encourage TERB through public shaming.

Thirdly, the findings of this study also suggested that tourism destination managers could emphasize the cultural and natural aspect of their destination, as it exerts complementary influences on TERB. Tourist destinations with a good destination image (both ecological and cultural) will stimulate tourists’ perception and interests and will induce their participation within the destination that may further elicit emotional attachment and subsequent responsible behavior, such as TERB, in the destination [84,85]. For example, at Australia’s Great Barrier Reef, many tourists are drawn to its stunning natural beauty and are also aware of the importance of protecting the delicate ecosystem. As a result, they are often willing to pay extra for eco-friendly tours and accommodation that prioritize sustainability and environmental protection. Moreover, the promotion and management of TERB benefits beyond sustainable tourism development, but more importantly into the area of ecological and sustainable social progression. As such, DMOs should foster a cultural and natural oriented destination image so to cue tourists’ emotion of awe and eventually their TERB for the destination.

Finally, the findings of this study will contribute to the promotion of sustainable development in tourist destinations, thereby making a significant contribution to the realization of a series of the United Nation’s Sustainable Development Goals (SDGs). This constitutes the social implications of this research. The tourism industry has become a vital economic sector for many underdeveloped countries and regions. The development of tourism has promoted their industrial development and infrastructure construction (SDG 9), provided numerous job opportunities (SDG 8), and helped the people in these countries and regions to escape poverty (SDG 1) and hunger (SDG 2). However, the tourist appeal of these underdeveloped countries and regions often stems from their unique natural environment and cultural customs. Protecting the natural environment and maintaining the uniqueness of the culture are the foundation and guarantee of their tourism development. This study provides insights for the DMOs of these countries and regions to promote local sustainable tourism development from the perspective of tourist behavior. By regulating tourist behavior, it promotes responsible production and consumption in the tourism industry (SDG 12), protects local aquatic and terrestrial organisms (SDG 14, 15), and preserves the authenticity of the culture. Thus, the sustainable development of the tourism industry aids in the realization of the SDGs.

## 8. Limitation and Future Research

This research posed limitations and opportunities for future research. This study examined the interrelationship between environmental perception, awe, face consciousness, and environmentally responsible behaviors, revealing their mediated-moderated relationship. While contributory, as noted in a previous study [86], the sole reliance on quantitative study in this study may hinder in-depth insights in regards to the relationship results. For instance, a detailed explanation on how and why the moderating effect of face consciousness on the relationship between awe and TERB is achieved. Future studies could address this limitation by replicating this study using a mixed-method approach with the employment of both quantitative as well as qualitative methods to substantiate the current findings.

In addition, this research was conducted in Tibet, China which is filled with its plateau natural landscape, mysterious folk customs, and remote location and is regarded as a highly distinctive and unique tourist destination. Undoubtedly, destinations with similar characteristics, such as Mount Kinabalu in Malaysia or Oros Olympos in Greece, should be examined in future research with the replication of this study to provide further empirical evidence to the model.

Furthermore, it would be interesting for future research to include related concepts, such as emotional solidarity, local attachment, and individuals’ norms to the model. It should be noted that the model is not finite and should be improved via theoretical enhancement as well as phenomenon changes. The integration and addition of theories and concepts would add value not just the model but, more importantly, to the formation of tourists’ attitude towards the environment that are essential for the success of sustainable tourism development.

Finally, it would be interesting for future research to replicate this study at the same location with different groups of tourists, such as those who are Western-educated, industrialized, rich, and democratic, extending beyond the Chinese sample in Tibet. The data would allow for a comparison between groups in understanding the predictive power of face consciousness. The findings could add value to the formation of TERB, which is significant for destination sustainability.

## Figures and Tables

**Table 1:** Results of confirmatory factor analysis.

Variables	Standardized Factor Loading	CR	AVE	Cronbach’s Alpha
**Humanistic environment perception (HEP)**		0.859	0.506	0.840
HEP1	0.771			
HEP2	0.711			
HEP3	0.755			
HEP4	0.695			
HEP5	0.579			
HEP6	0.738			
**Natural environment perception (NEP)**		0.759	0.512	0.752
NEP1	0.713			
NEP2	0.749			
NEP3	0.684			
**Awe**		0.903	0.573	0.898
Awe1	0.695			
Awe2	0.601			
Awe3	0.756			
Awe4	0.830			
Awe5	0.862			
Awe6	0.774			
Awe7	0.752			
**Tourist environmentally responsible behavior (TERB**)		0.826	0.544	0.816
TERB1	0.633			
TERB2	0.761			
TERB3	0.808			
TERB4	0.737			
**Face consciousness (FC)**		0.950	0.729	0.949
FC1	0.837			
FC2	0.870			
FC3	0.834			
FC4	0.904			
FC5	0.899			
FC6	0.859			
FC7	0.768			

**Table 2:** Construct intercorrelation.

Variables	1	2	3	4	5
1. Humanistic environment perception	**0.711**				
2. Natural environment perception	0.692 **	**0.716**			
3. Awe	0.318 **	0.261 **	**0.757**		
4. Tourist environmentally responsible behavior	0.465 **	0.316 **	0.253 **	**0.738**	
5. Face consciousness	0.387 **	0.397 **	0.112 *	0.321 **	**0.854**

Note: * *p* < 0.05, ** *p* < 0.01. Bold diagonal elements denote the square root of the AVE for each construct.

**Table 3:** Results of moderated mediation analysis.

Variables	Mediators: Awe			Dependent Variable: TERB		
Model 1	β	SE	95% CI	β	SE	95% CI
HEP (X_1_)	0.414 **	0.058	0.300, 0.528	−0.369	0.243	−0.846, 0.108
Awe (M)				0.543 **	0.194	0.161, 0.924
FC (W)				−0.228	0.285	−0.788, 0.332
Inter_1: X_1_ × W				0.185 **	0.057	0.072, 0.298
Inter_2: M × W				−0.103 *	0.045	−0.191, −0.016
R^2^	0.101			0.274		
F	51			34		
**Model 2**	**β**	**SE**	**95% CI**	**β**	**SE**	**95% CI**
NEP (X_2_)	0.337 **	0.058	0.222, 0.451	−0.133	0.198	−0.522, 0.256
Awe (M)				0.571 **	0.201	0.176, 0.966
FC (W)				0.261	0.279	−0.287, 0.808
Inter_1: X_2_ × W				0.080	0.048	−0.015, 0.174
Inter_2: M × W				−0.098 *	0.046	−0.190, −0.007
R^2^	0.068			0.187		
F	33			21		

Note: HEP = Humanistic environmental perception; NEP = Natural environmental perception; FC = Face consciousness. N = 456; CI = confidence interval; SE = standard error; * *p* < 0.05; ** *p* < 0.01.

**Table 4:** Results of the index of moderated mediation effect for TERB at value of face consciousness.

Moderated Mediation Effect	Index of Moderated Mediation	Boots 95% CI	Supported?
H6a: HEP → (Awe × FC → TERB)	−0.043	−0.089, −0.002	No
H6b: NEP → (Awe × FC → TERB)	−0.033	−0.073, 0.006	No

Note: Arrows pointing to effects within parentheses indicate moderation of those effects. The index of moderated mediation is the test of moderated (or conditional) mediation for each of the separate mediation paths [80]. Boots 95% CI: Bias corrected 95% confidence interval based on 5000 bootstrap samples.

## Data Availability

The data presented in this study are available upon request from the corresponding author.

## Appendix A

**Table A1:** The items of the constructs and normality test.

No	Item	Mean	SD	Skewness	Kurtosis
**HEP**	Humanistic environment perception				
HEP1	The cultural landscape of Tibet is sacred and solemn	4.61	0.559	−1.169	0.810
HEP2	The cultural landscape of Tibet is colorful and mysterious	4.63	0.563	−1.449	2.324
HEP3	The cultural landscape of Tibet makes me feel the magic and powerful power of faith and culture	4.48	0.679	−1.205	1.169
HEP4	I think it will take a long time to create the cultural landscape of Tibet.	4.45	0.763	−1.367	1.612
HEP5	The cultural landscape of Tibet makes me feel small	4.15	0.972	−1.024	0.484
HEP6	The cultural landscape of Tibet reminds me to remain humble in the face of its beliefs and culture	4.46	0.772	−1.506	2.045
**NEP**	Natural environment perception				
NEP1	The natural landscape of Tibet makes me feel the magic and powerful power of nature	4.62	0.603	−1.700	3.832
NEP2	I think it will take a long time to create the natural landscape of Tibet	4.55	0.684	−1.573	2.647
NEP3	The natural landscape of Tibet reminds me to remain humble in the face of nature.	4.56	0.730	−1.850	3.683
**Awe**	Awe				
Awe1	Traveling in Tibet, I feel (bored–amazed).	4.21	0.896	−1.118	1.247
Awe2	Traveling in Tibet, I feel (calm–struck).	4.15	1.049	−1.327	1.370
Awe3	The experience of traveling in Tibet is (ordinary–unique).	4.31	0.941	−1.426	1.815
Awe4	The experience of traveling in Tibet is (unimpressive–unforgettable).	4.43	0.833	−1.466	1.912
Awe5	The experience of traveling in Tibet is (boring–amazing).	4.24	0.874	−0.926	0.345
Awe6	Here, I feel (disdainful–reverent).	4.39	0.824	−1.336	1.632
Awe7	Here, I feel (discouraged–inspired).	4.29	0.878	−1.138	0.951
**TERB**	Tourist environmentally responsible behavior				
TERB1	I will report any environmental pollution or damage to the Tibetan authorities.	4.21	0.863	−0.926	0.554
TERB2	When I see litter, I will pick it up and put it in the trash bin.	4.38	0.743	−0.924	0.080
TERB3	If there are clean-up and environmental protection activities in Tibet, I am willing to participate.	4.33	0.788	−0.920	0.191
TERB4	I will persuade my travel companions (if any) to protect Tibet’s ecological environment.	4.55	0.617	−1.080	0.390
**FC**	Face consciousness				
	When I or my companions damage/pollute the environment in Tibet, I think:				
FC1	It may damage the image of myself in the eyes of others.	4.38	0.798	−1.447	2.463
FC2	It may cause others to question my moral level.	4.40	0.797	−1.490	2.577
FC3	It may embarrass you in front of others.	4.16	0.909	−1.031	0.829
FC4	It may make others think that my character is flawed.	4.24	0.908	−1.163	0.993
FC5	It may make others think that my quality is low.	4.30	0.863	−1.374	2.012
FC6	These behaviors reflect to some extent the difference in quality between me and others.	4.27	0.863	−1.323	1.914
FC7	These behaviors are not conducive to maintaining good interpersonal relationships with others.	4.24	0.911	−1.337	1.847
